# Inhibition of Rac1 GTPase activity affects porcine oocyte maturation and early embryo development

**DOI:** 10.1038/srep34415

**Published:** 2016-10-03

**Authors:** Si-Jing Song, Qiao-Chu Wang, Ru-Xia Jia, Xiang-Shun Cui, Nam-Hyung Kim, Shao-Chen Sun

**Affiliations:** 1 College of Animal Science and Technology, Nanjing Agricultural University, Nanjing 210095, China; 2 Department of Animal Sciences, Chungbuk National University, Cheongju 361-763, Korea

## Abstract

Mammalian oocyte asymmetric division relies on the eccentric positioning of the spindle, resulting in the polar body formation. Small signaling G protein Rac1 is a member of GTPases, which regulates a diverse array of cellular events, including the control of cell growth, cytoskeletal reorganization, and the activation of protein kinases. However, effects of Rac1 on the porcine oocyte maturation and early embryo development are not fully understood. In present study we investigated the role of Rac1 in oocyte maturation and embryo cleavage. We first found that Rac1 localized at the cortex of the porcine oocytes, and disrupting the Rac1 activities by treating with NSC 23766 led to the failure of polar body emission. In addition, a majority of treated oocytes exhibited abnormal spindle morphology, indicating that Rac1 may involve into porcine oocyte spindle formation. This might be due to the regulation of Rac1 on MAPK, since p-MAPK expression decreased after NSC 23766 treatments. Moreover, we found that the position of most meiotic spindles in treated oocytes were away from the cortex, indicating the roles of Rac1 on meiotic spindle positioning. Our results also showed that inhibition of Rac1 activity caused the failure of early embryo development. Therefore, our study showed the critical roles of Rac1 GTPase on porcine oocyte maturation and early embryo cleavage.

The mature mammalian oocyte is highly polarized and undergoes asymmetrical cell division because spindle migrates to the oocyte cortex to ensure the extrusion of small polar body in meiosis I (MI), which is essential for the generation of the large egg^1^. Spindle, chromosomes, and actin filaments are involved in the process of polar body extruding^2^,^3^. After the spindle formation, the chromosomes segregate and actin filaments (F-actin) together with myosin-II assemble into a circular structure along the cell equator, called the contractile ring in many eukaryotic cells. And then the polar body is extruded^4^. During this process, the meiotic spindle assembles around metaphase chromosomes and then moves peripherally to the cortex in an actin filament-dependent process^5^,^6^. The asymmetry results from oocyte polarization, which includes spindle positioning, migration and cortical reorganization, and this process is critical for fertilization and the retention of maternal components for early embryo development^7^. The early reproductive events start with folliculogenesis and end with blastocyst implantation into the uterine endometrium^8^. After fertilization, a zygote undergoes a first cleavage into two cells that is under maternal regulation^9^. And then the embryos undergo 4-cell, 8-cell, morula and blastocyst stage before implantation. *In vitro* embryo production is an effective tool to study early embryonic development in mammals, providing a condition to observe the state of embryos under microscope.

Rho family, one of the small monomeric G protein families, has been attended as a regulator of actin-based cytoskeletal rearrangements^10^. Major members of Rho family are Rho, Rac, and Cdc42. Rho GTPase controlled many cytoskeleton-dependent processes^11^,^12^, including cell motility, cell adhesion, cytokinesis, cell morphology, and cell growth^13^,^14^, which is known to regulate the assembly of focal adhesions and actin stress fibers in response to growth factors^15^. Cdc42, RhoA, and Rac1 were all demonstrated to be critical for oocyte spindle positioning: RhoA plays versatile roles in many aspects of cell function such as stress fiber formation, cytokinesis, and cell polarization^16^, which was recently considered to regulate cytoskeleton dynamics by affecting actin filament assembling and spindle formatting^17^. Another member of Rho family Cdc42 was also considered to be a key regulator of cytoskeleton and polarity, which is essential for meiotic maturation and oocyte asymmetry^2^. RhoA and Cdc42 was confirmed associate with actin assembly in sea urchin and Xenopus egg^18^. And several studies showed that RhoA, Rac1, and Cdc42 were found to have similar functions, such as in human esoghageal cancer cell^19^. Therefore, small GTPases have important roles in process of cytokinesis.

Rac1, as a characterized Rac subfamily member, can regulate a large variety of different functions, including the organization of the actin cytoskeleton, cell migration, cell cycle progression, and cell survival through engagement of specific effectors^14^. In human colorectal cancer LoVo cells, deletions of Rac1 affect the F-actin of cytoskeleton, arrest cell cycle in G0/G1 and induce apoptosis^20^. As previous studies showed in migrating endothelial cells (EC), Rac1-AuroraA-MCAK signaling pathway mediates polarization and directional migration^21^. In mouse oocyte, Rac1 was shown as a main regulator of oocyte polarization and meiotic division. A Rac-GEF triggered a localized activation of Rac in the oocyte cortex as a result of spindle migration and in return affected polar body emission during the meiosis II (MII) arrest^22^.

NSC 23766 is a specific inhibitor of the binding and activation of Rac GTPase. *In vitro* studies have shown that NSC 23766 inhibits Rac1 binding and activation via Rac-specific GEF Trio or Tiam 1 without altering RhoA or CDC42 binding or activation^23^,^24^. In addition, membrane type 1-matrix metalloproteinases (MT-1MMP) expression in CB CD34+ cells has been reported to decrease in the presence of NSC 23766^25^. *Silvetacompressa* investigations indicate that NSC 23766 depolarizes endomembrane cycling, altered polar adhesive secretion, and tip growth^26^.

Although members of Rho family were confirmed to participate to regulate actin filaments assembling and polarization indifferent species or models, such as in mouse oocyte^22^, whether and how Rac1 affects porcine oocyte maturation and embryo development were still unknown. Hypothesis was proposed that Rac1 GTPase involved in cytoskeleton-related processes in oocyte maturation and embryo development. In this study, we investigated the effects of Rac1 on porcine oocytes maturation and embryos development by treating with Rac1 GTPase inhibitor NSC 23766, and our result showed that the treated oocytes failed to extrude polar bodies and develop to blastocyst, indicating the important roles of Rac1 in porcine oocyte and embryos.

## Results

### Rac1 localization in porcine oocytes

**Figure 1:**
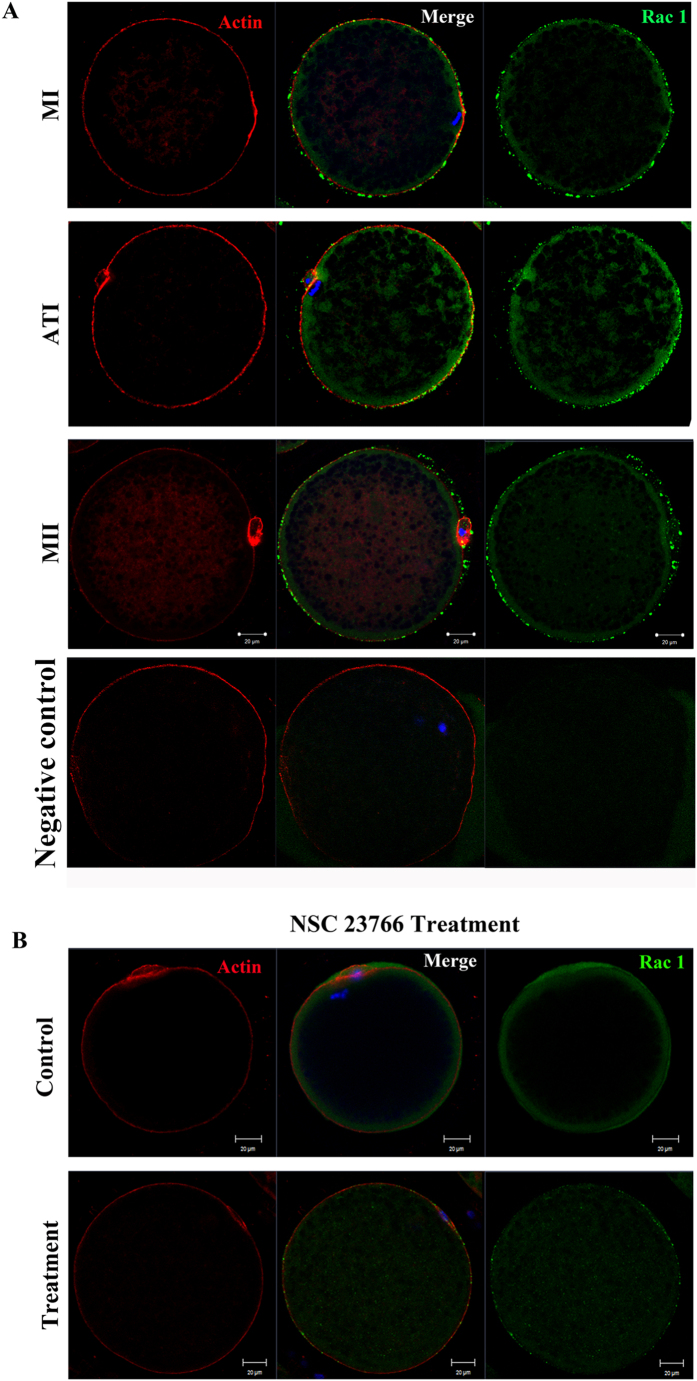
Rac1 protein localization in porcine oocytes. (**A**) Subcellular Rac1 localization at MI, ATI, MII stage of porcine oocyte meiotic maturation. Rac1 distributed all over the cortex during three stages. Blue, chromatin; red, Actin; green, Rac1. Bar = 20 μm. (**B**) Treatment group by Rac1 inhibitor, NSC 23766, and immunofluorescent staining for co-localization of Rac1 and Actin in oocytes. The immunofluorescent intensity is shown to decrease in treatment group. Blue, chromatin; red, Actin; green, Rac1. Bar = 20 μm.

We first examined the sub-cellular localization of Rac1 at different stages during meiotic maturation of porcine oocytes by immunofluorecent staining. We separately cultured oocytes for 25 h and 44 h to confirm the localization of Rac1 at meiosis I (MI) and Meiosis II (MII) stages. As shown in the Fig. 1A, Rac1 mainly distributed near the cortex of porcine oocytes during porcine oocyte meiotic maturation. By using double immunofluorecent staining we found the localization of Rac1 was similar with actin. Next we treated the oocytes with NSC 23766, and as shown in Fig. 1B, we found that the fluorescence intensity of Rac1 in the treatment group was significantly decreased, indicating that NSC 23766 could inhibit the activity or the distribution of Rac1 GTPase.

### Inhibition of Rac1 activity leads to polar body extrusion defects *in vitro*

**Figure 2:**
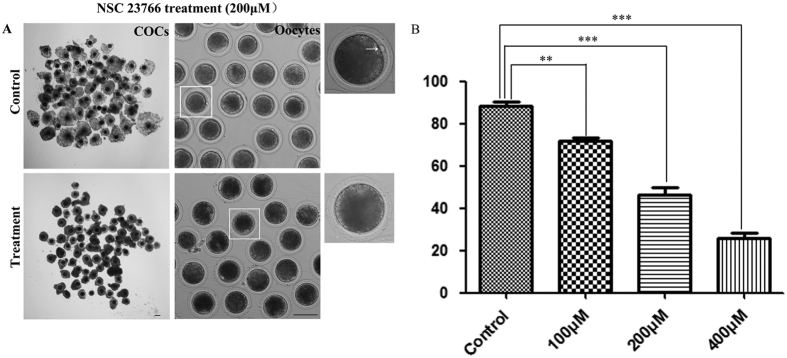
Rac1 inhibition effect on porcine oocyte maturation. (**A**) For COCs, the expansion of the peripheral layers cumulus achieved to more than in control group while it became progressively poor in NSC 23766-treated COCs; For oocytes, most oocytes extruded a first polar body (indicated by white arrow) in the control group whereas a great amount of oocytes failed to extrude the first polar body in treatment group. Bar = 120 μm. (**B**) Rac1 inhibition results in a decreased rate of first polar body extrusion among COCs. The effect of NSC 23766 on maturing oocytes was clearly dose dependent. The most suitable concentration for use with COCs was 200 μM.

To study the functions of Rac1 during porcine oocyte meiosis, we inhibited Rac1 activity by NSC 23766 and set 4 groups of different concentration. As shown in Fig. 2A, the expansion of the peripheral layers cumulus achieved to multiple layers in control group, whereas it became progressively poor in NSC 23766 treated COCs. Moreover, most control oocytes had extruded small polar bodies and were arrested at the MII stage, but NSC 23766 treatment caused polar body extrusion defects, leading to a lower rate of polar body extrusion. As a result, Rac1 inhibition reduced polar body extrusion in a dose-dependent manner (Fig. 2B). NSC 23766 treatment effectively inhibited polar body emission at concentration of 100 μM. Our results showed that 88.28 ± 5.18% (n = 249 COCs) of control oocytes extruded polar bodies while rates of polar body extrusion with 100 μM, 200 μM and 400 μM NSC 23766 treatment were significantly reduced to 72.18 ± 2.93% (*p* < 0.05; n = 235 COCs), 46.33 ± 9.99% (*p* < 0.01; n = 227 COCs) and 25.76 ± 5.44% (*p* < 0.01; n = 213 COCs), respectively (Fig. 2B).

### Inhibition of Rac1 activity affects meiotic spindle morphology

**Figure 3:**
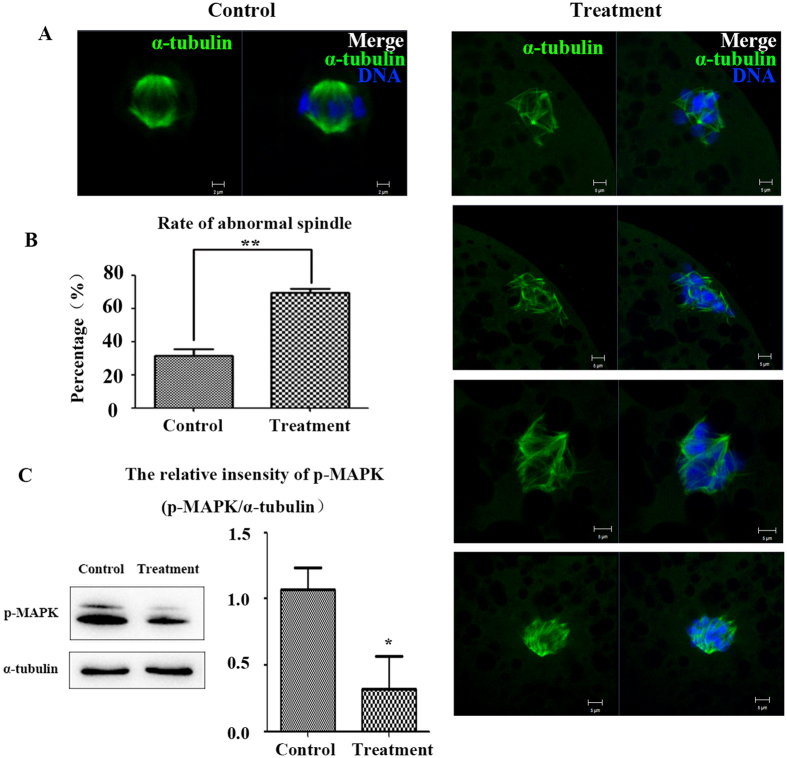
Rac1 inhibition affects formation of spindles. (**A**) Meiotic spindle morphology in MI oocytes. Control oocytes had morphologically normal spindles, whereas spindles were abnormal in inhibitor treated oocytes. Green, α-tubulin; blue, chromatin. Bar = 2 μm. (**B**)Rates of abnormal spindles. For NSC 23766 treated oocytes, the percentage of abnormal spindle morphology was significant higher than that of controls (*P* < 0.01). (**C**) p-MAPK expression was significantly reduced (*P* < 0.05) after inhibiting Rac1 activity in porcine oocytes.

For a further understanding of the roles of Rac1 in porcine oocyte, we examined spindle morphology after disrupting Rac1 activity. By culturing for 26 h, control oocytes in the MI stage had typical MI spindles (Fig. 3A). However, various disrupted spindle morphology was detected in Rac1-inhibited oocytes. To confirm this, the percentages of disrupted spindle morphology were counted. Normally, the spindle has two poles and regular “spindle” shape (Fig. 3A), while the disrupted spindle morphology showed several “non-spindle” shapes, such as multiple poles, enlarged poles, elongated poles, and none-pole. In the control group, the percentage of morphologically abnormal spindles was only 31.55 ± 7.01% (n = 51); while the percentage significantly increased to 69.28 ± 4.49% (n = 47; *p* < 0.01) for 200 μM NSC 23766 treated oocytes (Fig. 3B). Therefore, these results indicated that Rac1 was involved in meiotic spindle organization.

To determine the mechanism of Rac1 for meiotic spindle assembling, western blot was performed. Figure 3C and the supplementary information showed that p-MAPK protein expression was significantly reduced after NSC 23766 treatment. The relative intensity of p-MAPK protein expression (p-MAPK/α-tubulin) indicated the significant decrease for p-MAPK expression (NSC 23766-treated oocytes: 0.32 ± 0.35/1.07 ± 0.23; *p* < 0.05; n = 130). In conclusion, Rac1 might be essential for the expression of p-MAPK, which further regulated spindle organization during meiosis.

### Inhibition of Rac1 activity affects meiotic spindle position

**Figure 4:**
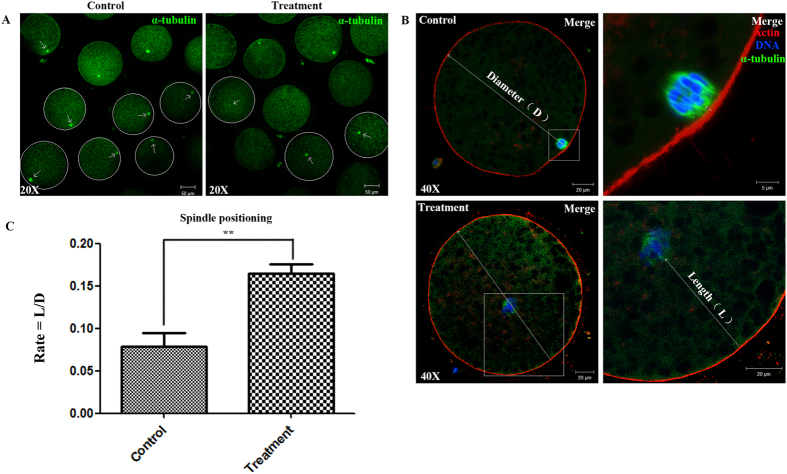
Rac1 inhibition affects spindle positioning in porcine oocytes. (**A**) Spindle localization in oocytes. For controls, spindles located peripherally, whereas they were nearly centrally located in NSC 23766 treated oocytes. Bar = 50 μm. Arrow indicates the position of the meiotic spindle. (**B**) Enlarged images besides showed the distances from spindles to the cortex. And we calculated the rate of length and diameter in a porcine oocyte to analysis. (**C**) In the control group, the spindles in most oocytes migrated to the cortex, whereas in the NSC 23766 treated group, the spindles of most oocytes remained at the center of the cytoplasm. The rate is significantly different (*P* < 0.01).

We next investigated spindle positioning and cortical reorganization. We chose the oocytes with flat spindle when scanning the sample, which means the spindle angle is horizontal, ensuring that the distance between spindle and cortex is the “correct” distance and avoiding the angle issue. After culture for 26 h, the locations of spindles could be observed under confocal fluorescence microscope. Compared to treatment group, a majority of spindles seem to have shorter distance to cortex in control group, while most spindles localized at the center of the oocyte in treatment group (Fig. 4A).

We then measured the distance between spindle and cortex in oocytes, and we got a value of rate by comparing it to diameter of the oocyte (Diagram, Fig. 4B). As shown in Fig. 4C, in the control group, this average rate was 0.078 ± 0.033 (n = 29), whereas in the treatment group, this rate was 0.164 ± 0.023 (n = 26); this rate was significantly higher than that in the control group (*p* < 0.05). These results indicated that disrupting Rac1 activity leaded to a failure of spindle formation and positioning and further disrupted polar body extrusion in porcine oocytes.

### Inhibition of Rac1 activity affects porcine early embryo development

**Figure 5:**
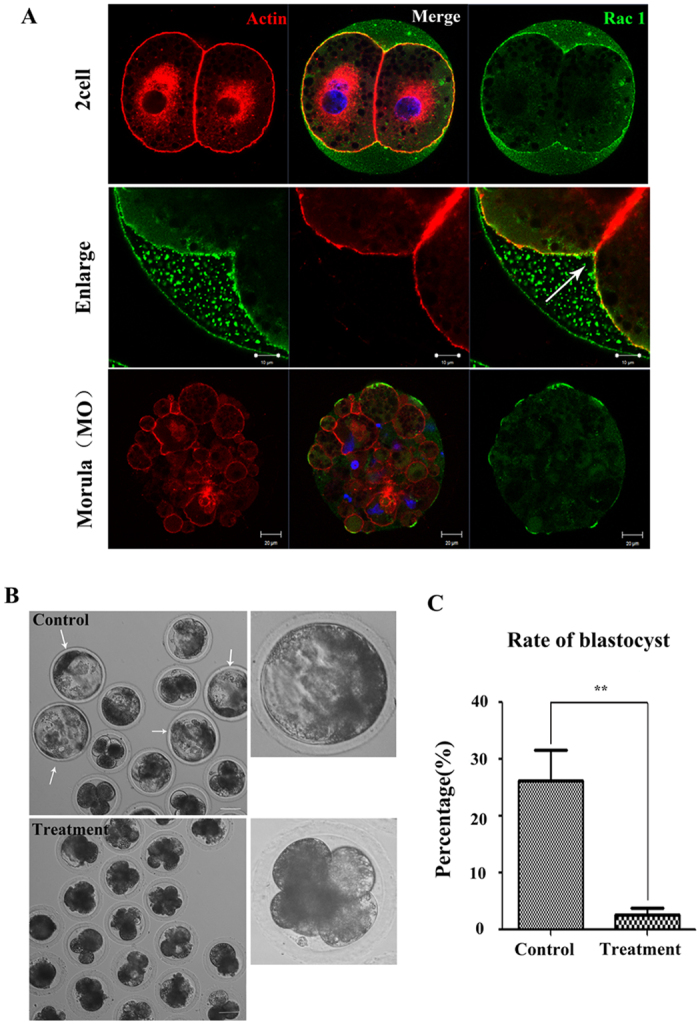
(**A**) Rac1 protein localization in parthenogenetically activated oocytes, and cleaving embryos. Rac1 protein sub-cellular localization during porcine parthenogenetic embryo development. Rac1 co-localized with actin from the 2-cell to morula stage. Arrow indicates the localization of Rac1 in embryos. Blue, chromatin; green, Rac1; red, actin. Bar = 20 μm. (**B**) Morphology of embryos derived from oocytes treated with Rac1 inhibitor. Rac1 inhibition resulted in aberrant morphology embryos. gray, DIC. Bar = 20 mm. (**C**) Rates of blastocyst after NSC 23766 treatment. The percentage of blastocyst was significantly decreased for treated oocytes (*P* < 0.01).

We also examined the expression of Rac1 in porcine embryos. Parthenogenetically activated oocytes were utilized. As shown in Fig. 5A, Rac1 appeared to be enriched in the cortex region, which is similar to the distribution of cortical actin filament layers at 2-cell stage. After compaction, Rac1 was concentrated at the periphery of the morula embryos.

To investigate the roles of Rac1 on embryo development, parthenotes were immediately cultured *in vitro* in the medium that was supplemented with NSC 23766 to monitor their ability to undergo cleavage divisions. A remarkable difference was detected at the blastocyst stage that most embryos could not maturate to blastocyst stage after Rac1 inhibition (Fig. 5B). According to Fig. 5C, for controls, 26.09 ± 9.51% (n = 107) of MII oocytes developed to the blastocyst stage while the percentage of the treatment group significantly declined to only 2.44 ± 2.38% (n = 98; *p* < 0.05).

## Discussion

Mammalian meiotic maturation is a process of asymmetric cytokinesis which is a unique characteristic of meiotic divisions in mammalian oocytes, producing oocytes with the small polar body size. Previous studies suggested that polarized Rac activity plays essential roles during meiosis, including the regulation of spindle dynamics, cortical anchoring of the MII spindle, and the completion of meiosis^22^,^27^. In this study, we showed that Rac1 played important roles in regulating maturation of porcine oocytes and embryos. And the results indicated that inhibiting Rac1 activity had influences on polar body extrusion, spindle positioning and spindle organization.

We first examined whether Rac1 was expressed in porcine oocytes and embryos. Our results showed that Rac1 exhibited specific localizations in both porcine oocytes and embryos, in particular, Rac1 accumulated exclusively at the cortex. The localization pattern of Rac1 in porcine oocytes and embryos was similar with actin filaments. Similar localization pattern was reported in several GTPases in oocytes. The large GTPase Dynamin 2 was shown to be localized at the cortex and around the spindles of oocytes, which is determined to regulate actin-mediated spindle migration in mouse oocytes^28^. Additionally, at MI, ATI, MII stages, Arp2/3 complex was concentrated primarily in the cortex of the oocyte, and Arp2/3 was also proved to regulate spindle formation and polar body extrusion in mouse oocytes^29^. And several studies showed that RhoA^17^ and CDC42^30^ had similar localization and regulated spindle formation. Therefore, we proposed a hypothesis that Rac1 might regulate spindle dynamics-related process in porcine oocytes and embryos. To prove our hypothesis, we disrupted Rac1 activity, which resulted in the cumulus expansion defects, the failure of oocyte polar body extrusion and embryo cleavage. Good cumulus expansion is one feature for the maturation of mammalian oocytes^31^. These findings proved that Rac1 inhibition affected the maturation of porcine oocytes and embryo development.

To further investigate the roles of Rac1 in porcine oocytes, we examined the meiotic spindle morphology. Meiotic spindles are composed of microtubules and are important for chromosome alignment and separation of maternal chromosomes during fertilization. During meiotic maturation, the meiotic spindle assembles around the centrally positioned metaphase chromosomes and then migrates to the cortex of the oocyte^32^. We found that a big proportion of abnormal spindle in the treatment group oocytes which was disrupted by NSC 23766. In previous research, members of small GTPase have been shown to be involved in the process of spindle assembling. Ran GTPase was proved to be essential to spindle assembly^33^,^34^, which mediates chromatin signaling to control cortical polarity during polar body extrusion in mouse oocytes^12^. Meanwhile RhoA^35^ as an upstream of FMNL1 was reported to regulate phosphorylates MAPK for spindle formation in mouse oocyte meiosis. In our study we showed that the Rac1 GTPase also regulated spindle organization in porcine oocytes. Together with previous study, these results indicated that small GTPases were widely involved into oocyte meiotic spindle formation. While we also showed that the effects of Rac1 on meiotic spindle might be due to its regulation on p-MAPK expression in porcine oocytes, since MAPK was shown to regulate meiotic spindle formation in oocytes^32^,^36^. Several previous studies showed the relationship between Rac1 and MAPK in many other species^37^,^38^,^39^. Together with these previous studies, our results indicated the conserved roles of Rac1 on MAPK in different models. Taken together, our results indicated that Rac1 played an important role in spindle formation.

We next examined spindle positioning during oocyte meiotic maturation. Accurate positioning of spindles is a critical aspect and step of oocyte cell division^40^. Meiotic chromosomes have been shown to induce cortical reorganization via a yet unexplained “at distance” effect^41^. A recent study confirmed that an actin flow drove spindles to migrate from the central to cortical area of oocytes, which resulted in oocyte asymmetric division^42^. Rac1 regulates polarized microtubule growth dynamics through Aurora A in migrating cells^21^. Meanwhile, Rac1 was found in mouse oocyte to regulate spindle anchoring during the metaphase II arrest^22^. Moreover, WAVE-2^43^ and Arp2/3 complex^29^ were essential for actin filaments assembly to affect spindle positioning in oocyte, and Rac1 is confirmed as an upstream molecule of WAVE-2/Arp2/3 pathway^44^,^45^. Our results showed that those oocytes treated with NSC 23766 exhibited spindle positioning defects, which resulted in centrally arrested spindles, leading to features of oocyte polarization after inhibiting Rac1 treatment. Similar results were found in the other small GTPases like Rho GTPases, RhoA, Rac and Cdc42^46^, for example, Rho GTPases affected the migration of spindle, spindle positioning and polar body extrusion during mouse and porcine oocyte meiosis^17^,^30^. Moreover, Rac1 were detected in mouse oocyte to regulate meiotic spindle stability and polarization^22^. Thus, our results indicated that Rac1 participates in porcine oocyte polar body extrusion through regulating spindle positioning.

In summary, our results showed that Rac1 regulated spindle formation and positioning, which involved into porcine oocyte maturation and embryo cleavage.

## Materials and Methods

### Antibodies and Chemicals

A rabbit polyclonal anti-Rac1 (C-14) antibody and Rac1 inhibitor (NSC 23766) which specifically inhibits the binding and activation of RacGTPase was from Santa Cruz (Santa Cruz, CA, USA). Phalloidin-TRITC and Alexa Fluor488 antibodies were from Invitrogen (Carlsbad, CA, USA) and mouse monoclonal anti-α-tubulin-FITC antibody were from Sigma-Aldrich (St. Louis, MO, USA).

Basic maturation culture medium was tissue culture medium (TCM-199) (St. Louis, MO, USA). PBS was obtained from Life Technologies (Invitrogen, Carlsbad, CA, USA). Porcine follicular fluid (pFF) was aspirated from 3–6 mm follicles of ovaries. The fluid was collected and centrifuged at 2000 rpm for 30 min at 4 °C, then stored at −20 °C under sterile conditions before use.

### Cell Culture and Development

Animal use was conducted in accordance with the Animal Research Institute Committee guidelines of Nanjing Agricultural University, China. This study was specifically approved by the Committee of Animal Research Institute, Nanjing Agricultural University, China. We collected porcine ovaries from prepubertal gilts at a local slaughter house and took them to the laboratory in sterile saline (0.9% NaCl) which contains 75 mg penicillin G/ml and 50 mg streptomycin sulfate/ml maintained at 37 °C. Then choose the medium sized follicles (3–6 mm in diameter) of ovaries to extract cumulus-oocyte complexes (COCs) with a needle and a 1.2 mm diameter syringe. The COCs surrounded by a compact cumulus mass and also with uniform ooplasm were isolated from the cellular debris.

Each 500 uL maturation medium for cell culturing contains the solution of TCM-199 with 0.1%polyvinyl alcohol, 0.57 mM cysteine, 0.91 mM sodium pyruvate, 3.05 mM glucose, 75 mg/L of penicillin, 50 mg/Lof streptomycin, 10% (v/v) pig follicular fluid (pFF), 10 ng/ml epidermal growth factor (EGF), 10 IU PMSG/ml, 10 IU hCG/ml. And grouped COCs were cultured in a 4-well dish (NUNC) at 38.5 °C in 5% CO2. To assess granulose-free oocytes, we treated it with 0.02% (w/v) hyaluronidase (in TCM-199) for 5 min at 38 °C, which process can separate oocyte from surrounding cumulus cells, so that we observed whether oocytes were alive to continue the next procession.

### Parthenogenetic activation to produce embryo *in vitro*

After culturing COCs for 44 h, the separated cumulus-free porcine oocyte can be observed if it extruded polar bodies under a Nikon Inverted microscope, which we selected to product the parthenogenetic activation embryos *in vitro*. The denuded MII oocytes were placed in a chamber filled with activation medium (0.3 M mannitol, 0.05 mM CaCl2 and 0.1 mM MgCl2 and 0.1% BSA) for 3 min. About 10 oocytes were pull into an electrode wire chamber to be activated each time with a set-up of an electrical pulse of 0.63 kV/cm, 80 ms, and 1 DC. Then, activated oocytes were washed 3 times and finally transferred to 500 ml of porcine zygote medium 3 (PZM-3), then embryos were maintained at 38.5 °C in 5% CO2. In this experiment, we counted the rate of cleavage at 24 h for 2-cell embryos, 48 h for 4-cell embryos and 96 h for blastocyst.

### NSC 23766 treatment

Rac1 inhibitor, NSC 23766 (5 mg) was dissolved in water to a concentration of 100 mM for conservation. Then we diluted it to 100 μM, 200 μM and 400 μM for cell culture. COCs and embryos were cultured in this medium for different periods of time meanwhile the treatment group use water instead.

### Immunofluorescence staining and confocal microscopy

For immunostaining of Rac1, α-tubulin and actin, two groups of oocytes or embryos at various culture periods were fixed in 4% paraformaldehyde (in PBS) for 30 min at room temperature and the permeabilized in permeablization solution (1% Triton X-100 in PBS) for 8–12 h at room temperature. To suppress nonspecific binding of IgG, oocytes embryos were blocked in blocking buffer for 1 h at room temperature. For Rac1 staining, we transferred oocytes or embryos into dilution of rabbit polyclonal anti-Rac1 antibodies for 1:100 in blocking buffer at 4 °C overnight. After 3 washes (2 min each) in washing buffer (0.1% Tween 20 and 0.01% Triton X-100 in PBS), samples were labeled with 1:500 dilution Fluor 488goat-anti-rabbit IgG for 1 h at room temperature. All samples were co-stained with Hoechst 33342 for 10 min and then washed three times in washing buffer, also were co-stained with α-tubulin or Phalloidin-TRITC depending on co-localization.

Samples were mounted on glass slides and examined with a confocal laser-scanning microscope (Zeiss LSM700 META). At least 30 oocytes or embryos were examined for each experiment.

### Protein extraction and protein western blot analysis

A total of 100 porcine oocytes at MI stage were collected, places in Laemmli sample buffer (SDS sample buffer and 2-Mercaptoethanol) and boiled at 100 °C for 10 min. After being cooled on ice and centrifuged at 12000 × g for 4 min, samples were frozen at −20 °C until use. Proteins were separated by sodium dodecyl sulfate-polyacrylamide gel electrophoresis (SDS-PAGE) with a Criterion precast gel (Bio-Rad, Richmond, CA, USA). After electrophoretic separation, proteins were electrophoretically transferred onto a polyvinylidene fluoride membrane (Millipore, Billerica, MA) for 1.5 h at 100 V at 4 °C using a semidry blotting system. To avoid nonspecific binding, membranes were blocked 2.5 h with Tris-buffered saline (TBS) containing 0.1% (w/w) Tween 20 (TBST) and 5% (w/v) nonfat dry milk powder for 2 h at room temperature. The membrane was simultaneously incubated with rabbit polyclonal rabbit anti-p-MAPK (1:2000; Cell Signaling Technology, Danvers, MA, USA) primary antibodies at overnight 4 °C, respectively. Then, the incubation buffer was 2% BSA in TBST. Subsequently, after being washed 3 times in TBST (10 min each), membranes were treated in horseradish peroxidase (HRP) conjugated anti-rabbit IgG (1:2000; Cell Signaling Technology, Beverly, MA, USA) for 2 h at room temperate. Finally, the membranes were washed 3 times in TBST and then the specific proteins were visualized using chemiluminescence reagent (Millipore, Billerica, MA). Equal protein loading was confirmed by the detection of a-tubulin (rabbit monoclonal anti-a-tubulin antibody; 1:2000; Cell Signaling Technology, Danvers, MA, USA). This experiment was repeated at least 3 times using different samples.

### Statistical Analysis

For conclusion, three replicates were used for each treatment with results expressed as means ± SEMs. Statistical comparisons were made by Analysis of Variance (ANOVA), followed by Duncan multiple comparisons test. In a t-test, if a “*p*” value is less than 0.05, we considered it as significant.

Fluorescence intensity and western blot analysis used Image J (NIH) software with at least 10 samples to analyze for each experiment.

## Additional Information

**How to cite this article**: Song, S.-J. *et al.* Inhibition of Rac1 GTPase activity affects porcine oocyte maturation and early embryo development. *Sci. Rep.* **6**, 34415; doi: 10.1038/srep34415 (2016).

## Supplementary Material

Supplementary Information
