# Supplementary material for: Inhibition of Rac1 GTPase activity affects porcine oocyte maturation and early embryo development
Source: Sci Rep. 2016 Oct 3;6:34415. doi: 10.1038/srep34415 (PMC5046063; doi:10.1038/srep34415)

Inhibition of Rac1 GTPase activity affects porcine oocyte maturation and early embryo development

Si-Jing Song^1^, Qiao-Chu Wang^1^, Ru-Xia Jia^1^, Xiang-Shun Cui^2^, Nam-Hyung Kim^2^, Shao-Chen Sun^1^*

^1^College of Animal Science and Technology, Nanjing Agricultural University, Nanjing 210095, China.

^2^Department of Animal Sciences, Chungbuk National University, Cheongju 361-763, Korea.

**^*^Correspondence to:** Shao-Chen Sun, College of Animal Science and Technology, Nanjing Agricultural University, Nanjing, China. Tel/Fax: +86-25-84399092. E-mail: [sunsc@njau.edu.cn](mailto:sunsc@njau.edu.cn)

Supplementary figure: The original figure for the bands of p-MAPK (42/44 kDa), α-tubulin (52kDa) and the band marker for the western blot experiments.


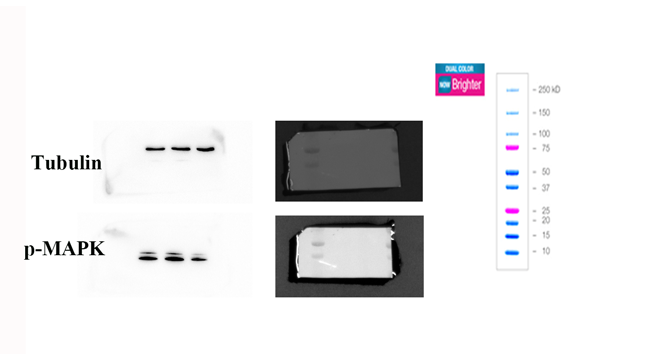

Supplement: Supplementary Information [file srep34415-s1.doc]
